# Charles- Bonnet Syndrome: a case review. The objective of this poster is to contribute a case to the existing series, and thus get closer to the knowledge of this clinical entity.

**DOI:** 10.1192/j.eurpsy.2023.1981

**Published:** 2023-07-19

**Authors:** L. Huerga García, I. Careno Baez, G. Oropeza Hernández, A. Marcos Rodrigo, C. Delgado Torres, G. Garriga Rocío

**Affiliations:** 1 Psychiatry; 2 Hospital Universitario Nuestra Señora de la Candelaria, Santa Cruz de Tenerife, Spain

## Abstract

**Introduction:**

Charles-Bonnet syndrome was described in 1760 by the Swiss philosopher Charles-Bonnet, who reported that his grandfather’s visual hallucinations were due to eye disease rather than mental illness.

It is characterized by the presence of visual hallucinations, which are usually complex and structured, in elderly patients with preserved cognitive status, significant deterioration in visual acuity and no evidence of associated psychiatric or neurological disease.

**Objectives:**

The objective of this poster is to contribute a case to the existing series, and thus get closer to the knowledge of this clinical entity.

**Methods:**

To review the case, a search was made in Pubmed with the terms hallucinations and Charles Bonnet’s Syndrome.

**Results:**

This is a 76-year-old man, in follow-up by the ophthalmology service in the context of bilateral cataract, which causes severe visual disturbance. He went to our hospital, accompanied by his wife, reporting that for some months he has had complex visual hallucinations of various animals, colors in space, as well as children playing around him. All this generates a lot of anxiety, although the patient makes adequate criticism of them.

The neurological examination performed was normal. The CT scan and laboratory tests were also within normal limits. Cognitive impairment was explored using the MMSE scale, which did not show any alteration. In addition, after a psychiatric evaluation, the patient does not meet the criteria for any disorder included in the DSM V. After reviewing the literature and taking into account the clinical picture described, the case is framed within a Charles-Bonnet syndrome.

Regarding the therapeutic plan carried out, it was decided to start treatment with Gabapentin up to a maximum dose of 900 mg/day, with a considerable improvement in the hallucinatory symptoms. In addition, given the repercussion at the affective level, especially with a predominance of anxious symptoms, it was decided to start sertraline at a dose of 50 mg/day, with an adequate therapeutic response.

**Conclusions:**

Charles-Bonnet syndrome refers to hallucinosis, generally of a visual nature, that appear in patients with a sensory deficit associated with the type of sensory-perceptive alteration. It is important to take it into account in the differential diagnosis of the elderly patient with hallucinosis. There is no established treatment, although neuroleptics, benzodiazepines, antidepressants and antiepileptics are used.

**Disclosure of Interest:**

None Declared

